# A review of the recycling of non-metallic fractions of printed circuit boards

**DOI:** 10.1186/2193-1801-2-521

**Published:** 2013-10-09

**Authors:** André Canal Marques, José-María Cabrera Marrero, Célia de Fraga Malfatti

**Affiliations:** Metallurgy Department (DEMET)/ PPGE3M, Federal University of Rio Grande do Sul, Porto Alegre, Brazil and UNISINOS, Porto Alegre, Brazil; ETSEIB-Department of Materials Science and Metallurgical Engineering, Universidad Politécnica de Catalunya, Barcelona, Spain; Fundacio CTM Centre Tecnologic, Materials Forming Area, Manresa, Spain; Metallurgy Department (DEMET)/ PPGE3M, Federal University of Rio Grande do Sul, Porto Alegre, Brazil

**Keywords:** Waste electrical and electronic equipment (WEEE), Non-metallic fractions (NMFs), Recycling, Printed circuit boards

## Abstract

There is a big waste generation nowadays due to the growing demand for innovation and the fact that more and more products have a reduced lifetime, increasing the volume of dumps and landfills. Currently, one of the segments of large volume is the technology waste, which reflects on the printed circuit boards (PCBs) that are the basis of the electronics industry. This type of waste disposal is difficult, given that recycling is complex and expensive, because of the diversity of existing materials and components, and their difficult separation process. Regarding the material involved in PCBs, there are metal fractions (MFs) and non-metallic fractions (NMFs), of which the recycling of NMFs is one of the most important and difficult processes, because they amount to about 70% of the weight of the PCB’s waste. In the present paper, a literature review of the recycling of non-metallic fractions (NMFs) has been carried out, showing different studies and guidelines regarding this type of recycling, emphasizing that this type of waste still lacks for further application.

## Introduction

As reported by several researchers (Cui 2003; Murugan 2008; Guo et al. 2009), the production of Electrical and Electronic Equipment (EEE) is one of the fastest growing sectors of manufacturing industry in the world, expecting a 3-5% increase per year. In parallel, there is a falling life expectancy for electronics, low recycling rates, large variability among the Waste Electrical and Electronic Equipment (WEEE) due to the continuous changes in product design, increased legal and illegal global trade of these products (Puckett et al. 2002; Brigden et al. 2005; Deutsche Umwelthilfe 2007; Wong 2007; Cobbing 2008; Williams et al. 2008; Sepúlveda 2010). This results in large amounts of electronic waste, which must be solved because it is becoming a major social problem and a threat to the environment (Brodersen et al. 1992; Lacoursiere 2005; He et al. 2006; Lee et al. 2007). And this is particularly true in which concerns to printed circuit boards (PCBs) that are the basis of the electronics industry.

Murugan (2008), Chancerel and Rotter (2009) pointed out that the rate of generation of WEEE is high worldwide and continues to increase, being one of the fastest and most growing waste flows. In 2002, it was estimated that the electronics occupied approximately 4% of municipal waste (Emery 2002) and in 2005 these items constituted 8% of municipal solid waste Widmer et al. (2005). Huisman et al. (2008) stated that, in 2007, the generation of waste amounted to 8.3-9.1 million tons per year, which corresponds to about 17 kg per capita per year. Recent statistics indicate that the total annual global volume of WEEE should soon reach 40 million tons (Unep 2005; Unu 2007). The proportion of WEEE (printed circuit boards waste) in electronic waste is about 3% (Bernardes et al. 1997; Basdere and Seliger 2003).

**Figure 1:**
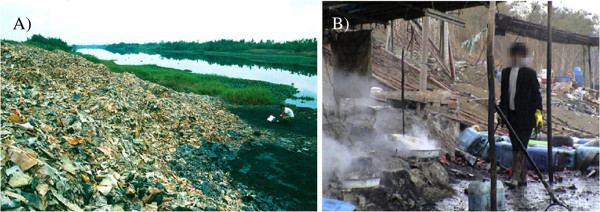
**Environmental Damages. A)** Dumping of circuit boards treated with acid and processing waste along River Lianjiang in China. **B)** Recovery of gold from PCB waste using acid baths Source: Ban 2002.

Sepúlveda (2010) said that used PCBs have attracted more attention of public and researchers, because abundant toxic materials including heavy metals and Brominated Flame Retardants (BFRs) can easily be found in them, and that these can cause enormous damage to the environment if not properly treated (seen in Figure 1) (Cui and Forssberg 2003 Aea Technology Environment 2004 Wang et al. 2005 Eps Canada 2006 Owens et al. 2007 Leung et al. 2008 Vasile 2008 Zhou and Qiu 2010; Sohaili et al. 2011 and 2012; Zeng et al. 2012).

According to Guo et al. (2009), in general, the components of PCB waste can be divided into metal fractions (MFs) and non-metallic fractions (NMFs). A lot of work is being undertaken to investigate every step of the recycling of PCB waste (Lee et al. 2000 and 2004; He et al. 2006), of which NMFs recycling is one of the most important and difficult ones.

The main economic driving force for the recycling of PCBs is the value of MFs contained in PCBs (Zheng et al. 2009). After being mechanically separated, recycled metals such as Cu, Al, Fe, Sn, Sb, Pb, etc., are sent for recovery operations and these processes are already mature. Yet the NMFs are landfilled or incinerated, which will cause waste of resources and potential environmental problems (Rotter, 2002; Jang and Townsend 2003; Hagelüken 2006; Owens et al. 2007; Huang et al. 2007; Janz et al. 2008).

Several authors (Richter et al. 1997; Menad et al., 1998; Riess et al. 2000; Vehlow et al. 2000; Cui and Forssberg, 2003; Ebert and Bahadir, 2003; Schlummer et al. 2007; Dimitrakakis, 2008; Taurino et al. 2010), argue that WEEE plastics contain brominated flame retardants (BFRs), including polybrominated biphenyls (PBB) and polybrominated diphenyl ethers (PBDEs) and the combustion of these NMFs induces the formation of highly toxic gases, the dibenzodioxins and polybrominated dibenzofurans and dioxins and furans (PBDD/Fs), besides the fact that glass fiber significantly reduces fuel efficiency.

With the rising costs of landfills and imminent legislation for recycling of resources, there is a great need to develop technologies for recycling nonmetallic materials of used PCBs. It is clear that, with the exception of hazardous substances, a large amount of valuable materials contained in WEEE becomes worthy of being recycled (Zhou and Qiu 2010). This paper presents a review of the current situation of recycling of NMFs, showing some kinds of recycling carried out with these types of materials.

## PCB waste compositions and characteristics of the NMFs

The choice of materials used to manufacture PCBs depends on the application, for example, bifunctional epoxy resins are suitable for simple circuit boards, double sided (Guo et al. 2009). Two common types of PCBs are made of glass fiber reinforced with epoxy resin (commercially referred to as FR-4) or cellulose paper reinforced with phenolic resin (FR-2). FR-4 type PCBs are used in high value EEE and FR-2 types are used in televisions and electronics (Lassen and Lokke 1999; Jawitz 1997; Hall and Williams 2007).

The components of a PCB generally include chips, connectors, capacitors, etc., each one manufactured with different materials (Bernardes et al. 1997; Wilkinson 2001). The material composition of the PCB is therefore quite complex and causes problems for the recycling of PCB waste. The typical composition of non-metallic fractions (NMFs) is: thermosetting resins (epoxy), fiberglass, plastic, reinforcement materials, additives and other BFRs and represents about 70 wt% of PCB waste. The metallic fractions is composed of copper ~ 16%, tin-lead ~ 4%, iron and ferrite ~ 3%, nickel ~ 2%, silver ~ 0.05%, ~ gold 0.03%, palladium ~ 0.01%, and so on (Sum, 1991; Iji and Yokoyoma 1997; Veit et al. 2002; Goosey and Kellner 2003; Li et al. 2004 Sohaili et al. (2011 and 2012).

As previously mentioned, the material compositions vary according to origin and type of PCB and waste separation process. In a study by Iji and Yokoyoma (1997), the NMFs consisted of glass fiber (65 wt%), cured epoxy resin (32 wt%), and impurities (copper: < 3 wt%, soldering alloys: < 0.1 wt%). In another study (Zheng et al. 2009), in which air classification was used as a separation process, the content was different as listed in Table 1.

**Table 1:** **Content of NMFs material from recycled PCB waste**

***Specimen***	***NMFs particle size***	***Cu content (wt%)***	***Glass fiber content (wt%)***	***Resin, etc. content (wt%)***
1	Fine	1.64	71.50	26.86
2	Medium	1.05	55.50	43.45
3	Coarse	0.48	46.00	53.52

Source: Adapted from Zheng et al. (2009).

## Physical recycling of non-metallic fractions from PCB waste

In general, the methods of recycling NMFs from PCBs can be summarized as physical recycling methods and chemical recycling methods. The physical recycling methods are adopted after the NMFs fractions of MFS and NMFs are separated from PCB waste using mechanical processing (Veit et al. 2006; Zeng et al. 2012). The mechanical processing for the separation of MFS and NMFs of PCB waste include shape separation, magnetic separation, separation based on electrical conductivity, density-based separation and corona electrostatic separation method, which have been well reviewed by Cui and Forssberg (2003), Huang et al. (2008) and Guo et al. (2009). The corona electrostatic separation (CES) for the MFs and the NMFs from PCB waste has been intensively studied by many researches (Li et al. 2007 and 2008; Lu et al. 2006 and 2008; Wu et al. 2008a and 2008b; Jiang et al. 2008a and 2008b; Sohaili et al. (2011 and 2012).

Franz (2002) reported that the use of non-metals for thermoplastics would be the perfect solution for recycling. Other authors argue that non-metals can be used as fillers for epoxy resin products such as paints, glues, agents of decoration and building materials (Iji and Yokoyoma 1995 and 1997; Li et al. 2004; Hong and Su 1996; Arya et al. 2002; Mou et al. 2007; Guo et al. 2009; Zheng et al. 2009). In fact, most researchers recycle NMFs as charges for thermoset resins and thermoplastic resins in considering the physical recycling methods. Although these applications are better than putting these residues in landfills or incineration, many improvements are still needed to promote such uses.

Mou et al. (2005) introduced new methods in which non-metals were used to make models, composite boards and related products. Non-metals were used for a kind of board with some additives, and also used in the production of phenolic molding compounds to replace the sawdust (Li et al. 2007; Rao et al. 2008).

The advantages of physical recycling methods is that the treatment is relatively simple, practical, investments in equipment and energy costs are low and the potential application of products made from recycled NMFs is diversified. However, the recycling of WEEE is just beginning (Cui and Forssberg 2003). The fundamental issue in the physical recycling of NMFs is how to use it in an effective, cheap and safe way for the different materials Guo et al. (2009). The recycling of NMFs is much more difficult than the routine waste recycling because the compositions of the NMFs are diverse, complex and even toxic.

### Application as reinforcing filler for thermoplastic resins

Polypropylene (PP) as one of the most important commodity polymers is widely used in various applications. Due to its good processability, recyclability and low cost, this polymer has found a wide range of applications in packaging, textiles, automotive and furniture industry (Liang 2002; Zebarjad 2003; Alcock et al. 2006). However, due to its low resistance, low modulus and high notch sensitivity, the utility of PP as an engineering thermoplastic is still limited. To expand the range of applications, the challenge of increasing the strength and modulus of PP has attracted great interest. The placement of fillers in PP with rigid inorganic particles is an effective, economical and convenient way to improve its strength and stiffness (Leong et al. 2004; Cho et al. 2006; Yang et al. 2007).

In general, the fillers for polymers have two functions: one is to reduce the cost of goods and the other is to improve product performance. Currently, the fillers with high performance play a key role in the field of high-tech materials. Therefore, how to take advantage of the fillers for polymer products is an important issue.

Zheng et al. (2009) performed mechanical tests showing that both tensile and flexural properties of non-metals/PP composites can be significantly improved by the addition of non-metals in PP. The maximum increment of tensile strength, elastic modulus, flexural strength and flexural modulus of PP composites was 28,4%, 62,9%, 87,8% and 133,0%, respectively. Up to 30% of non-metal in weight of recycled PCB waste can be added in PP composites without violating environmental laws.

### NMFs waste to replace wood flour in the production of phenolic molding composites (PMC)

According to Guo et al. (2009), phenolic resins are one of the oldest and most common thermoset resins. Phenolic molding composites (PMC) are produced with phenolic resin, acting as a binding agent, various fillers, gelling and dyes under high temperature and a certain pressure. Due to its relative low price, ease of fabrication, high mechanical strength, heat resistance and high dielectric strength, PMC is widely used and is being used in radios, kitchen appliances, and electronic switches. The growth in production of PMC in recent years greatly increased the need for wood flour, which is used as organic filler in molding compounds. With the depletion of timber resources and the increasing price of wood flour, it is a task for PMC producers to protect timber resources and reduce the cost of raw materials by finding alternative materials to wood flour. But there are few studies on the use of non-metals subtracted from PCB as a filler of PMC.

**Figure 2:**
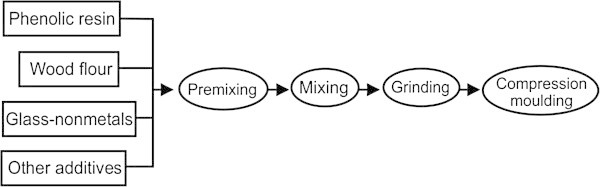
**PMC production flow chart.** Source: Rao et al. (2008).

The type of PCB waste used for Rao et al. (2008) was made of glass fiber reinforced with epoxy resin. The non-metals recovered from this type of PCB were used to replace wood flour for the production of PMC. The process of production of PMC is shown in Figure 2.

**Figure 3:**
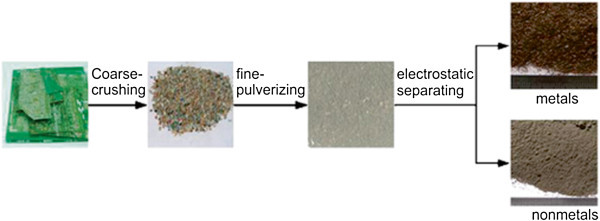
**Schematic illustration of grinding and electrostatic separation of PCB.** Source: Rao et al. (2008).

The residues of PCB were first pulverized in a process that consists of a coarse grinding stage and a stage of fine pulverization, using a cutting machine and a hammer mill. Then, an electrostatic separator was used to separate metals from non-metals (Li et al. 2007). The particles of copper and non-metallic materials, after two stages of crushing and corona electrostatic separation are shown in Figure 3. After being separated, the NMFs were selected by a vibrating screen.

**Figure 4:**
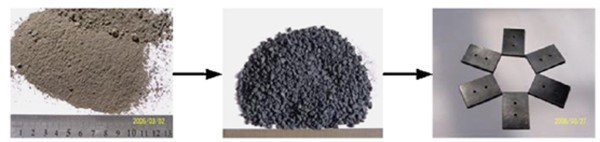
**Schematic illustration of glass-nonmetals of waste PCBs filling in the PMC.** Source: Rao et al. (2008).

According to Rao et al. (2008), to maintain the same relative density, the proportions of wood flour and talcum powder were adjusted. The powders at the end of the process were molded by compression into test samples using different molds, according to the corresponding standards. The specimens of PMC are shown in Figure 4.

From the standpoint of industrial application, a recycling ratio (the amount of NMFs in PMC) of 20% was more reasonable. All the results showed that the use of NMFs as filler in PMC represented a promising method to solve environmental pollution and reduce the cost of PMC, thus achieving the environmental and economic benefits.

### Fillers for building materials

**Figure 5:**
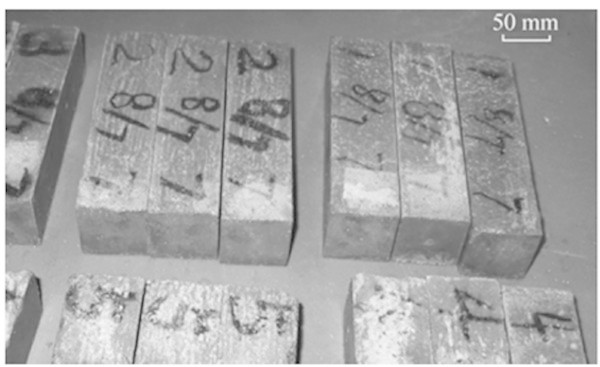
**Bricks made using recovered nonmetallic PCB material.** Source: Mou et al. (2007).

As stated by Mou et al. (2007), in the case of building materials, the mechanical strength under bending and compression forces is one of the most important property. Compared to the main concrete materials, i.e. cement, sand and water, non-metallic powder recovered from PCBs is lighter than cement and sand, has a finer granularity which makes the microstructure more reliable, and contains thicker glass fibers that improve the mechanical strength. The ideal ratio of non-metallic powders in construction materials should be carefully determined to maximize the flexural and compressive strength, being the key factor to find an optimum ratio of resin mix. Some bricks made from non-metal powder recovered from PCB are shown in Figure 5.

Niu and Li (2007) and Siddique et al. (2008) reviewed the use of recycled plastic in concrete, noting that NMFs can also, to some extent, be used with some effectiveness as a partial replacement of inorganic aggregates in concrete applications to reduce the dead weight of structures and increase the concrete properties such as compressive strength, tensile strength, modulus, impact resistance, abrasion resistance and permeability. Panyakapo and Panyakapo (2008) has reused thermoset plastic waste for lightweight concrete. His study indicated that the use of thermoset waste to produce lightweight concrete is a promising method. The NMFs are a mixture of waste thermosets, glass fibers and other components, and therefore, can replace the melamine waste to produce lightweight concrete. His results show that NMFs improves compressive strength and flexural strength of concrete if they are properly mixed.

However, according to Mou et al. (2007), the reuse of recovered non-metals as filler to make construction materials is feasible, but not very attractive, since improvements in bending strength and compressive strength are limited to less than 10% when compared to standard concrete and replaced materials in the original concrete, sand and cement, which are cheap and plentiful, so this substitution will not bring a good economic return. Thus, the ongoing investigation into the use of recycled PCB as building material simply intends to find some use for the recycled material even if it is a relatively small amount.

### Modeling material

There are many types of models used for decoration, mostly made of plaster, plastic or other materials (Mou et al. 2007). The resin powder has many similar or better physical properties than the cast, including lower weight, waterproof, easy to shape and mechanical strength (due to glass fibers), so it could, therefore, be used as a substitute for statues, ornaments and other models. Three different molding processes have been used to make models of NMFs. Models made of PCB non-metals and adhesive: Final results showed that all models were solid, but there was little mechanical strength. Models made of PCB non-metals and decorative cement: The PCB non-metallic material was used as the primary material for models designed as ornaments. Special decorative cement served as an adhesive. Models made of non-metallic powder, decorative cements and fillers: Some fillers were added to the cement powder and decorative cement to enhance the features of the model. When compared to the use of PCB non-metallic powder filler for building materials, the use of models, while not perfect, has better prospects since the recovered material is not only filler, but the main materials, to reuse what has more value.

### Composite boards made of PCB non-metallic materials

**Figure 6:**
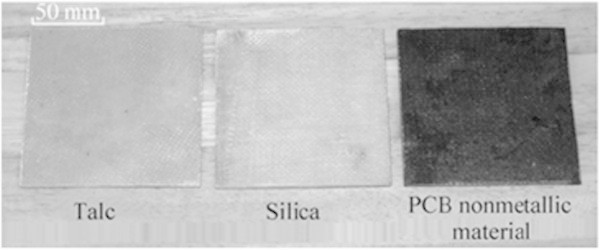
**Composite boards made from talc, silica, and the PCB nonmetallic material.** Source: Mou et al. 2007.

According to Mou et al. (2007) and Sohaili et al. (2011 and 2012) using non-metallic material recovered from PCBs to make composite boards and related products is very attractive. The composite boards are widely used in many areas, including cars, furniture, entertainment equipment, and decorative materials. The most interesting aspect of making composite boards out of NMFs of PCBs is the potential economic benefit. In general, products made from composite boards are high-value ones with high profit margins. A wide variety of products can be made from composite boards for various applications. The main components in the composite boards are listed in Table 2. Composite boards made of talc, silica, and PCB non-metallic materials are shown in Figure 6.

**Table 2:** **Key components in composite boards**

***Ingredients***	***Ratio (wt. %)***
**Glass fiber cloth**	**30-45**
**Epoxy resin**	**35-40**
**Fillers**	**5**
**PCB nonmetals**	**15-30**

Source: Mou et al. 2007.

During the production process, the boards made from recovered non-metallic materials were more easily shaped and flattened than the boards of talc and silica. The mixing time was also significantly reduced and the mixture was more uniform and reliable than the mixture of silica and talc. These improvements can be attributed to the good affinity between the recovered material and the resin-based adhesive (Mou et al. 2007).

### Specific products made of non-metallic materials of PCBs

Mou et al. (2007) and Sohaili et al. (2011 and 2012) also pointed out that the recovered non-metallic material from PCBs can be better used to make products that support higher bending stresses due to its excellent resistance to bending. The technology has been used in two typical products.

(1) Sewer grates

**Figure 7:**
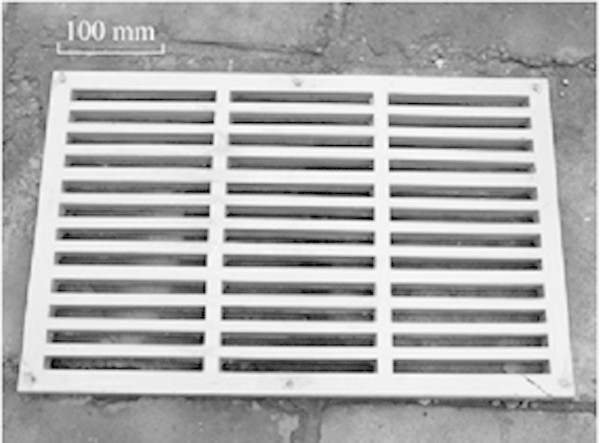
**Sewer grate made from recovered PCB nonmetallic material.** Source: Mou et al. 2007.

According to Mou et al. (2007), China needs about 4 million sewer grates a year, which have been made of composite materials (Figure 7), and the steel fiber concrete and fiberglass reinforced with plastic (FRP) are the two most common materials. The main advantages of these are lower cost and better mechanical resistance, especially resistance to bending.

(2) Surfboat in amusement park

**Figure 8:**
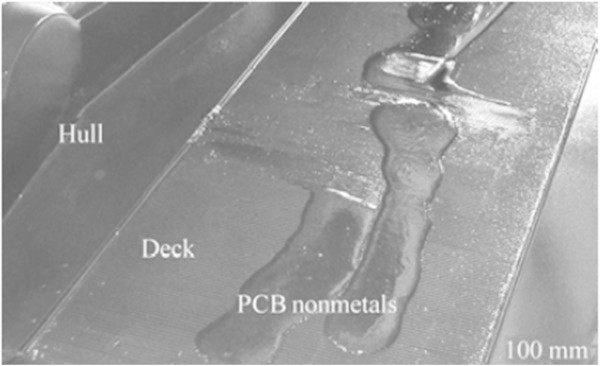
**Deck with PCB non-metallic material.** Source: Mou et al. (2007).

Mou et al. (2007) points out that the company “Beijing SBL Amusement Equipment Co., Ltd” manufactures boats, among other products for amusement parks. The deck of the boat is subject to great flexion pressures. The composite plate can be effectively used on the floor and the body of boats. The first batch of boats made with PCB non-metallic materials has undergone performance tests and is being used at the Shijingshan Amusement Park in Beijing, Figure 8.

### Recycling the NMFs as a modifier for viscoelastic materials

Iji and Yokoyoma (1995) has conducted research using recovered NMFs as a filler for viscoelastic materials made of epoxy resin compounds, comparing the powder with common fillers, such as calcium carbonate, talc and silica. The epoxy resin compound consists of an epoxy resin matrix (48 wt%), a hardener (20 wt%) and a filler (32 wt%). The NMFs found improved mechanical strength and thermal expansion properties of the epoxy resin mold, being NMFs more effective than talc, calcium carbonate and silica. This improvement in properties was probably due to the compatibility between the NMFs and the epoxy resin matrix, and also because of the inclusion of fiberglass. The viscosity of the compound with the NMFs was comparable to the compound with powder. The bond strength of epoxy resin with casts of NMFs was sufficient, that is, it was almost equal to the mold with calcium carbonate, talc or silica.

Asphalts are widely used in paving roads. But the modified asphalt is highly susceptible to changes in temperature due to their rheological properties. Great emphasis was placed on improving the performance of asphalt (Sengoz et al. 2008), and the use of polymers as asphalt modifiers is considered a relatively new and interesting change, because it involves two important aspects: the use of waste materials (low cost) and the enhancement of the asphalt properties. The glass fibers and powder resins contained in the NMFs can be used to reinforce the asphalt. The glass fibers and powder resins contained in the asphalt NMFs reinforce the asphalt by the effect of composition, and the addition of these materials reduces the cost of asphalt, and, therefore, there is an environmental and economical gain.

## Chemical recycling of NMFs

According to Guo et al. (2009), chemical recycling is the decomposition of polymeric waste into monomers or into some useful chemicals through chemical reactions. The main objective of the methods of chemical recycling is to convert the polymers present in the NMFs into chemical inputs or fuels. Compared to physical methods of recycling, chemical recycling methods have the advantage of converting BFR into monomers and removing heavy metals left in the waste (Long et al. 2010; Xiu and Zhang 2010; Ni et al. 2012; Weia et al. 2012; Ke et al. 2013; Yang et al. 2013; Zeng et al. 2013).

The chemical recycling methods (Chiang et al. 2007) include pyrolysis, gasification, process of depolymerization using supercritical fluids and hydrogenolytic degradation. Hazardous substances in NMFs are mainly BFRs (tetrabrombisphenyl-A (TBBA), etc.) and traces of heavy metals (lead, chromium, cadmium, mercury, etc.) (Dimitrakakis, 2008). The removal and treatment of BFRs and heavy metals contained in NMFs is a definite method to eliminate pollution caused by hazardous substances. Chemical recycling is considered by Guo et al. (2009) the most effective method to take full advantage of all the elements and eliminate all hazardous and toxic components contained in the NMFs. Unfortunately, the studies are limited and relatively few data are available.

The chemical recycling trend of NMFs of PCB waste is to improve the advantages in relation to physical recycling of NMFs to offset the rising cost of chemical recycling methods (Guo 2009). The refining of the products (gas and oil) is included in the chemical recycling process, however, all searches are concentrated on the separation and improvement processes, and the refining process can be done with conventional methods of refining in chemical plants.

### Recycling of NMFs from PCB waste by pyrolysis process

Pyrolysis is a chemical recycling technique that has been widely studied as a method of recycling synthetic polymers, including polymers that are mixed with glass fibers. Pyrolysis of polymers leads to the formation of gases, oils and chars that can be used as chemical feed stocks or raw material for fuels. In addition, if the temperature is high enough, the pyrolysis process will melt the soldering alloys used to attach electrical components to printed circuit boards. The combination of removal and recovery of the organic fraction of printed circuit boards and solder removal should help the separation of the metallic components of the organic material (Hall and Williams, 2007).

Hall and Williams (2007) investigated the pyrolysis of PCB residues from computer, televisions and cell phones in a fixed bed reactor, in order to separate and recover the organic and metallic materials. In their study, a selection of printed circuit boards was pyrolysed at 800°C and the pyrolysis products were analyzed. The pyrolysed computer PCB formed an average of 68,9% of weight of waste, 22,7% of weight of oil and 4,7% of gas in weight. The pyrolysis oils contained high concentrations of phenol, 4-(1-methylethyl) phenol and p-hydroxyphenol as well as bisphenol A, tetrabromobisphenol A, methyl phenols and bromophenols. The pyrolysis oils also contained significant concentrations of organo-phosphates and a series of tetrabromobisphenol A products, from the pyrolysis, were also identified.

In this study the pyrolysis residues were very fragile and the organic fractions, fiberglass and metallic fractions can be easily separated and the electrical components can be easily removed from the remains of printed circuit boards. The ash in the residue consisted mainly of copper, calcium, iron, nickel, zinc and aluminum, as well as the lowest concentration of valuable metals such as gallium, bismuth, silver and gold. Silver was present in particularly high concentrations. ICP-MS and SEM EDX have also identified many other metals in the ash. The pyrolysis gases consisted mainly of CO_2_ and CO, but all C1-C4 alkanes and alkenes were present as well as some inorganic halogens.

Due to the persistence and toxicity of PBDD/Fs (polybrominated dibenzodioxins and dibenzofurans), a combined method of control to inhibit both PCDD/Fs (Polychlorinated dibenzodioxins) and PBDD/Fs is of great importance for the recycling of NMFs from PCB waste by pyrolysis process. Lai et al. (2007) studied the inhibition of the formation of PBDD/Fs from the pyrolysis of PCB waste. The efficiency can be improved and the pyrolysis temperature can be reduced by the addition of suitable catalysts during pyrolysis. According to Guo et al. (2009), it is possible to prevent the formation of PBDD/F with optimal treatment and the addition of CaO on pyrolysis of waste from PCB. The oils produced by NMFs through chemical recycling methods must be refined before practical use, but the cost may be higher than the conventional routes to prepare oil and petrochemicals, so it can be difficult to convince petrochemical companies to gauge their interest in the process.

### Recycling of NMFs from PCB waste by gasification process

The main objective of gasification processes in the processing of polymer wastes is the generation of synthesis gas (CO, H_2_). Possible by-products in these processes are CO_2_, H_2_O, CH_4_ and soot. The reaction temperature range is up to 1600°C at a pressure of 150 bar. The correct choice of the gasification process itself can also be decisive. The most valuable product is a synthesis gas rich in hydrogen with a high proportion of reactive components (not inert) and only minor impurities. This product is a valuable raw material for the synthesis of methanol. It can be used to generate heat and electricity (Sasse and Emig, 1998).

Yamawaki (2003) studied the gasification technology of the recycling of WEEE plastics containing brominated flame retardants, which gives a reference to the recycling of NMFs from PBC by gasification processing. This work showed that high temperature treatment and shock cooling suppressed the emission of PBDDs/PBDFs to a very low level and that, when the cooling was slow after the high temperature treatment, the emission of PBDDs/PBDFs reached values 2300–4300 times higher than those obtained after the rapid shock cooling. This means that there was regeneration of PBDDs/PBDFs during slow cooling. The results show that the gasification of WEEE plastics containing brominated flame retardants can be achieved to avoid the generation of brominated dioxins and prevent the regeneration of brominated dioxins. Therefore, the gasification of NMFs from PCB waste can be considered a method of chemical recycling that can prevent the generation of brominated dioxins. However, the relevant investigation at this point is just beginning.

### Recycling of NMFs of PCB waste by depolymerization in supercritical fluids

A supercritical fluid is any substance at a temperature and pressure above its thermodynamic critical point. Supercritical fluids (SCFs) and especially supercritical water (SCW) are the potential means for the recycling of fibers and resins, because they may be inexpensive, recyclable, non-toxic and relatively easy to handle means of reactions. Under supercritical conditions, water, organic compounds and gases are completely miscible. In addition, supercritical water is emerging as a useful chemical medium that could provide better conditions for a variety of chemical reactions, including the destruction of hazardous waste (Guo et al. 2009).

Chien et al. (2000) used SCW to oxidize PCB waste, converting the resins contained in NMFs from waste to CO_2_, H_2_O, NaBr, etc. In fact, besides being used for hydrothermal treatment of PCB waste oxidation, SCW can be used to recycle NMFs of PCB waste by depolymerization process, because it is an excellent hydrolysis reagent. Tagaya et al. (2004) studied the decomposition reactions of epoxy resin in SCW. In the reaction of epoxy resin, the yield of identified products reached 10% for the reaction at 703K more than 1 h. The results suggest that water played an important role not only as a physically stable means at high temperatures, but also as a chemical reagent.

### NMFs recycling of PCB waste for hydrogenolytic degradation

The hydrogenolytic degradation is an innovative recycling technique for the recycling of raw materials based on thermosetting resins, although there are few reported studies. The research by Braun et al. (2001) gave a good reference for recycling of thermosets by hydrogenolytic degradation. In this investigation, it was shown that many branched polymers, especially epoxy resins, can be liquefied by transfer hydrogenation with various hydrogen donors. Reticulated epoxy resin with phthalic anhydride, phenolic resin and melamine resin were used as materials in their study. The most important products identified were bisphenol A and its fragments of phenol and p-isopropylphenol and phthalic anhydride and its fragments of benzoic acid and benzene.

With the method described, Braun et al. (2001) attempted to liquefy a raw material for circuit boards covered with copper foil on both sides and reinforced with 59,5% of fiber glass sheets. It can be seen that it is not necessary to grind this material before hydrogenolysis. The layers of fiberglass and copper foil were recovered with very little contamination.

## Conclusions

One of the main considerations in physical recycling of NMFs of PCB waste is using the NMFs as a safe and effective filler for different composites with matrix of thermoset and thermoplastic resins. The advantages of the physical recycling methods is that the treatment is relatively simple, convenient, the cost of investment in equipment and energy is low and the potential application of products made from recycled NMFs is diversified.

In turn, the main objective of the methods of chemical recycling is to convert the polymers present in the NMFs in inventory supply of chemicals or fuels. Compared with the physical one, chemical recycling methods have the advantages of converting BFRs in monomers and remove the heavy metals in the waste. The trend of the chemical recycling of NMFs of PCB waste is to make the best of advantages over physical recycling of the NMFs to offset the rising cost of chemical recycling methods.

It is clearly noted that NMFs of PCB waste must be recycled in a sustainable process. The combustion of NMFs will form highly toxic PBDD/Fs substances while the landfill of NMFs will lead to secondary pollution caused by heavy metals and BFRs leaching to groundwater. Thus, there is much work to be done to develop methods of physical or chemical recycling. However, research on this topic is just beginning and the challenges caused by technical and economic feasibility should not be underestimated.

Currently research recycling of both metal fractions (MFs) and non-metallic fractions (NMFs) are developing but with more emphasis on MFs. We believe that in the future recycling NMFSs will become a major focus of research on recycling of printed circuit boards. One of the biggest problems is that few companies have these technologies. We believe that one of the points to improve this context is that companies must be connect with universities and research centers, enabling technologies that are under development to become viable soon because often recycling technologies not keep pace with development new products and technologies that companies put on the market.
